# Psychosocial environment for the integrated education opportunities of the disabled in Lithuania

**DOI:** 10.1186/1471-2458-6-305

**Published:** 2006-12-18

**Authors:** Laimute Samsoniene, Algirdas Juozulynas, Gene Surkiene, Konstancija Jankauskiene, Aloyza Lukšiene

**Affiliations:** 1 Health and Sport Center, Vilnius University, Vilnius, Lithuania; 2 Institute of Experimental and Clinical Medicine, Vilnius University, Vilnius, Lithuania; 3 Institute of Public Health, Vilnius University, Vilnius, Lithuania; 4 Department of Physiology, Kaunas University of Medicine, Kaunas, Lithuania

## Abstract

**Background:**

The policy of the diminution of the social isolation of the disabled is the main objective of the strategy of the EU new policy concerning the disabled. Lithuanian society faces this objective as well. For this reason, this study aiming at providing the theoretical basis for and predicting the possible psycho-social environment in an integrated education system, as well as at the evaluation of the reasons for the formation of a positive approach to the disabled, is especially relevant, since it creates the prerequisites for the optimisation of the process of the integration of disabled schoolchildren into the general system of education.

**Method:**

The sample of the study consisted of 2471 children from the same schools: not integrated (1958), integrated (126) and special schools (382). Empirical methods: questionnaire poll, comparative analysis. The statistical analysis was carried out using *SAS*.

**Results:**

Our study showed that the majority of schoolchildren without disabilities and disabled schoolchildren have positive intentions for interpersonal interactions (>82%) and positive emotions (>69%) independently of the discrepant character of interpersonal contacts, different conditions of education and family life, and despite of low level of knowledge.

**Conclusion:**

The results of the study confirmed positive intentions for interpersonal interaction between disabled schoolchildren and schoolchildren without disabilities, as well as a positive character of emotions, and disprove the unsound myth of the opponents of the social integration of the disabled stating that disabled children in comprehensive schools would undoubtedly experience offence from their peers without disabilities.

## Background

It is thought that socialization is most successful in societies with simple division of labor and minimal distribution of knowledge [1]. Each member of the society knows who he/she is and who the others are. An individual becomes the member of the society when he/she realizes that he/she is living in the same world as people around him/her. An individual has to begin adjusting the motives of his/her behaviour to other society members, and begins constantly identifying him/herself and the others. However, there is a question of whether the individual agrees with his/her allocated position and role in the society [2].

The ideology of the individual medical approach to the disabled that is predominant in Lithuania contradicts the EU culture of socio-educational approach to the disabled, and causes legal policy contradictions between the healthcare system that promotes the stagnant rehabilitation approach to the disabled, and the educational system that has adopted the progressive educational approach [3].

Social interaction is unequivocally important to an individual. According to Lemme, social relationships must be evaluated in the wide social and historical context. They not only provide positive emotions, stimulate our self-awareness, and satisfy our cognitive needs, but can also result in emotional risk. Social relationships, health, and mortality are closely interrelated. Social isolation results in the deterioration of health, and poor health limits social contacts [4,5].

Our respondents learned and lived in specialized educational establishments, separately from their families. Their socialization took place under the predomination of external control that stimulates the child's obedience and values collective communication. Lithuanian scientists [6-9] also presented similar conclusions in their writings stating that children's separation from their families, and education in specialized educational establishments impedes the accomplishment of educational goals (one of the main goals of comprehensive education is the development of an independent personality capable of responsibly creating his/her life). In a specialized school, children create a distorted worldview, and therefore they wander away from the real life. In addition to that, the society thus alienates itself from the disabled as well [9].

The aim of our study was to evaluate the attitudes of disabled schoolchildren and their peers without disability towards each other and towards the integration of the disabled in the education system.

## Methods

The study was performed during 1999–2003. The research was conducted in 1999–2003. The permission of the Committee of Ethics has not been received as the Law of Biomedical Research was adopted only on January 1, 2001. The authors of the study got approval for their work in the Special Education Sector from the Ministry of Education and Science of the Republic of Lithuania. The participants of the study were schoolchildren aged 9–12, and 13 years. The oral consent was obtained from every participant of the study. The criterion of moral development played a crucial role in selecting the age of schoolchildren. Our interest focused on the early adolescence, when the morality of social rules is established, and adolescence, when a teenager follows the principles of his/her indisputable rights and personal ethics [6,9]. The pilot study was performed in the school of the integrated type, where 150 respondents – schoolchildren – were interviewed. Later on the same study was repeated in Vilnius, Kaunas, and Prienai schools. Comprehensive schools in Vilnius were chosen at random, whereas institutions of special education in Vilnius, Kaunas, and Prienai were selected according to the disability form, vision, hearing, motion or combined. The research was conducted anonymously, and the respondents filled the questionnaire independently. Of 2700 questionnaires distributed among schoolchildren, 91.6 percent were filled and handed back. The studied sample was represented by 2471 respondents – groups of schoolchildren from integrated and non-integrated comprehensive schools as well as from schools with special education. The homogeneity of the respondents was determined by the taught subject, and the homogeneity of schoolchildren – by their age and sex. In specialized schools, the type of disability of schoolchildren determined the heterogeneity of the groups.

Simple methods were applied in the research. According to Sultive and Ulrich [10], when dealing with the integration problems of the disabled, the applied statistical methods should be as simple as possible. Moreover, acquired statistical data are characteristic only of the participants of the study.

The empirical methods applied were questionnaire poll and comparative analysis.

Statistical data analysis was performed when applying the statistical methods. To compare the groups, Wilcoxon's Two-Sample Test was chosen. Spearman's correlation coefficient (r) was applied in the analysis of the correlative sample data relations. The significance level p < 0.05 was chosen for the verification of statistical hypotheses. In order to determine the reliability of the questionnaire data, Cronbach's alpha data reliability procedure was used (for schoolchildren without disabilities, α = 0.73 – reliable, and for schoolchildren with disabilities, α = 0.64 – reliable).

With a view to investigate the multidimensional attitude of the respondents towards each other as well as their attitude towards the integration process in the area of education, two types of authentic questionnaires were used for schoolchildren with and without disabilities. The questionnaire was composed of questions like *Has anybody talked or explained to you about the disabled people? *followed by fixed-choice *answers: 1. Never, 2. *Yes, sometimes, 3. Frequently. The answers were evaluated 1 to 3 points based on Ternary scale [Additional file 9,10]. The pilot study was performed among all age groups of the integrated school type. There were no difficulties encountered while filling the questionnaires. The reliability of the questionnaires was satisfactory.

A questionnaire for schoolchildren without disabilities was composed of the following components: a behavioural component of attitude (a desire to communicate with the disabled schoolmates occasionally, during spare time, and at school, and readiness to help them in a conflict situation or in daily living needs), a sensual component, and the index of knowledge (the level of knowledge and the need for knowledge).

A questionnaire for the schoolchildren with disabilities was composed of the following components: the relation to the schoolmates without disabilities (a desire to relate to them separately, during spare time, at school, to communicate at home, or in a conflict situation), the relation to other disabled (interpersonal communication, relation, and self-expression), sensual components of the attitude of the schoolchildren with disabilities towards their schoolmates with and without disabilities (emotions towards each other, self-worth and self-image of the disabled), and the index of the knowledge of the schoolchildren with disabilities (the level of knowledge and the need for knowledge).

According to Berger and Luckmann [1], the reality during the initial state of the child's socialization is perceived as inevitable. During this stage, internalization is based on emotions for as long as the individual is operating in the everyday world. However, when the borders of this world widen, marginal situations of human experience start to threaten the individual's internalization process, and this period of socialization becomes an unnatural one. For this reason we think that it is important to clarify a person's emotional attitude towards surrounding people in the aspects of these marginal situations of a child's experience.

## Results

**Figure 1:**
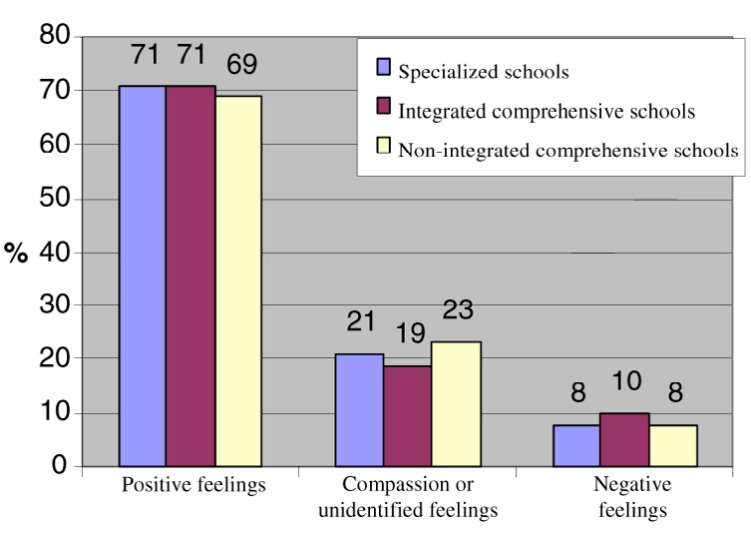
**The attitudes of schoolchildren towards reciprocal feelings**. The distribution of the percentage of schoolchildren without disabilities (integrated and non-integrated comprehensive schools) and schoolchildren with disabilities (specialized schools) according to the results of the emotional (positive, compassion or unidentified, negative feelings) components of reciprocal attitudes (HSAD, DSAH>^1^) in relation to the type of the school.

The attitudes of schoolchildren with and without disabilities towards reciprocal feelings were evaluated on the basis of the results of the investigation of the emotional components of the attitude of schoolchildren without disabilities towards the disabled ones (HSAD), and the attitude of schoolchildren with disabilities towards their peers without disabilities (DSAH-1) (see Figure 1). The obtained findings showed that the absolute majority (ca. 70 percent) of respondents experienced positive reciprocal feelings irrespectively of whether schoolchildren without disabilities were learning at an integrated or a non-integrated comprehensive school (p > 0.05). Only a small proportion of the respondents expressed negative feelings towards each other.

**Figure 2:**
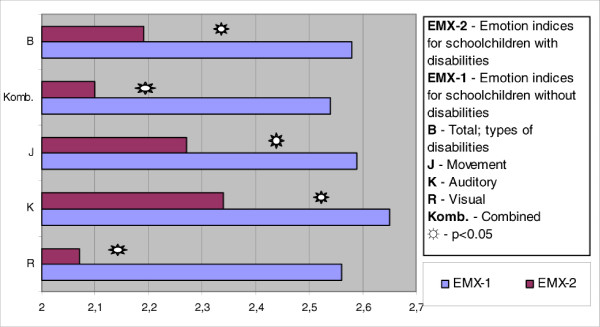
**The emotion indices of schoolchildren with disabilities by the type of disability**. The relationship between disabled schoolchildren's indices of emotions experienced towards their peers with and without disabilities, and the type of disability (visual, auditory, movement, combined).

**Figure 3:**
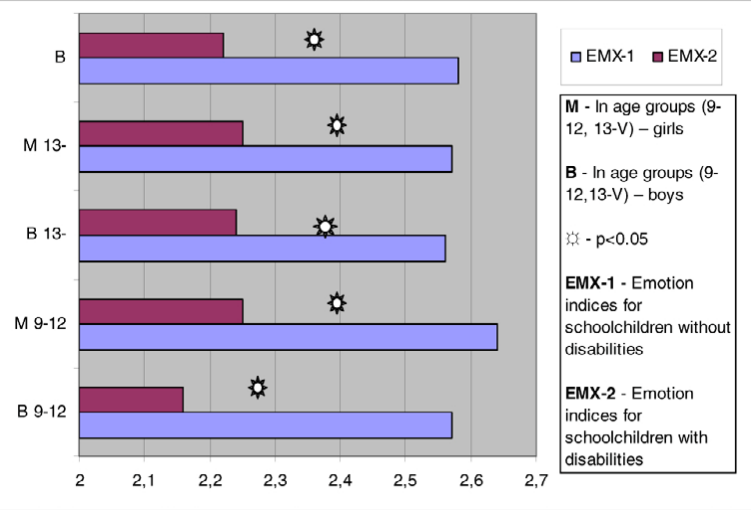
**The emotion indices of schoolchildren with disabilities by age and sex**. The relationship between disabled schoolchildren's indices of emotions experienced towards their peers with and without disabilities by age (9–12, 13-V) and sex (girls, boys).

The investigation of each component and indices of the disabled respondents' attitude with relation to age, sex, and the type of disability showed that schoolchildren with disabilities expressed much more positive feelings with respect to schoolchildren without disabilities than to the disabled ones. The analysis of the data on the relationship between the indices of the emotional components of the attitudes of schoolchildren with disabilities towards their peers without disabilities and towards disabled schoolchildren, and the type of disability [see Figure 2], age, and sex [see Figure 3] showed that in respondents with all the studied types of disability, the index of the emotions towards schoolchildren without disabilities (EMX-1) was reliably higher than the index of emotions experienced with respect to the disabled ones (EMX-2). The total emotion index with respect to the schoolchildren without disabilities was rather high (2.56 out of 3 pts.) with respect to either the type of disability, sex, or age, whereas the index of emotions experienced towards the disabled ones reached only 2.22 points.

Similar results were obtained having distributed the respondents with disabilities according to age and sex [see Figure 3]. In schoolchildren with disabilities, the index of emotions experienced towards their peers without disabilities (EMX-1) was reliably (p < 0.05) higher, compared to the index of emotions experienced towards the disabled peers (EMX-2). The total age- and sex-adjusted index of emotions experienced towards the disabled (position B) was reliably lower (p < 0.05), compared to the index of emotions experienced towards schoolchildren without disabilities, and amounted to 2.21 out of 3 points.

**Figure 4:**
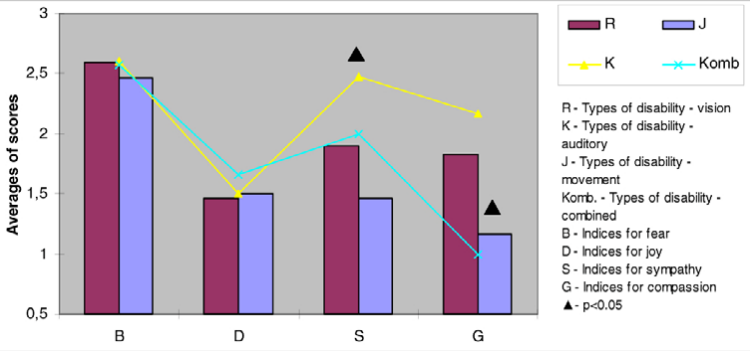
**The emotion component of schoolchildren with disabilities according to indices**. The indices (fear, joy, sympathy, compassion) of the disabled respondents' emotions towards their peers with disabilities, with relation to the type of disability (vision, movement, auditory, combined).

In order to determine why the emotions of schoolchildren with disabilities towards their disabled peers were less positive than those experienced towards schoolchildren without disabilities, we investigated the relationship of the EMX-2 indices with the type of disability [see Figure 4]. The obtained findings showed that schoolchildren with all the studied types of disability did not experience joy or fear when seeing their peers with disabilities (positions D and B). However, schoolchildren with auditory disorders manifested reliably greater sympathy and compassion towards their disabled peers (positions S, and G), compared to schoolchildren with other types of disability.

**Figure 5:**
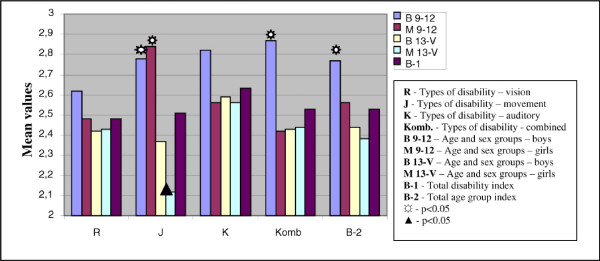
**The self-worth index of the schoolchildren with disabilities**. The relationship of the self-worth index of the schoolchildren with disabilities and the respondents' age (9–12, 13-old), sex (girls, and boys), and the type of disability(vision, movement, auditory, combined).

Having distributed the schoolchildren with disabilities into age groups according to sex and the type of disability, we noticed that older girls with movement disability manifested the highest levels of disability-related anxiety [see Figure 5], and their SX was reliably lower in all the studied groups (p < 0.05) according to age, sex, and the type of disability. Meanwhile, the SX of younger boys with combined, auditory, or movement disability was reliably (p < 0.05) more positive, compared to that among older girls or boys (positions J, K, and Komb.). According to our findings, the SX of respondents with vision disability was not dependent on either age, sex, or the type of disability (position R).

**Figure 6:**
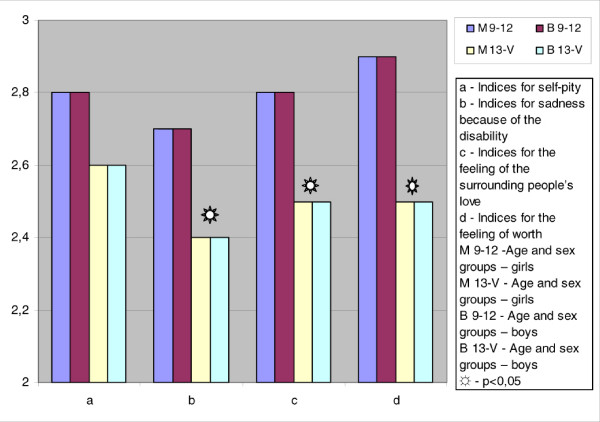
**The self-worth index of schoolchildren with disabilities by age and sex**. The relationship of the values of the respondents' self-worth index (self-pity, sadness because of the disability, the feeling of the surrounding people's love, the feeling of worth) with their age and sex (girls, boys).

The relationship of the disabled respondents' self-worth index (SX) of the emotional component of attitudes with respect to their disability, and the respondents' age and sex is presented in [see Figure 6]. According to our findings, SX indices reliably (p < 0.05) decreased with age (positions b, c, and d), whereas the respondents' sex did not have any influence on them.

**Figure 7:**
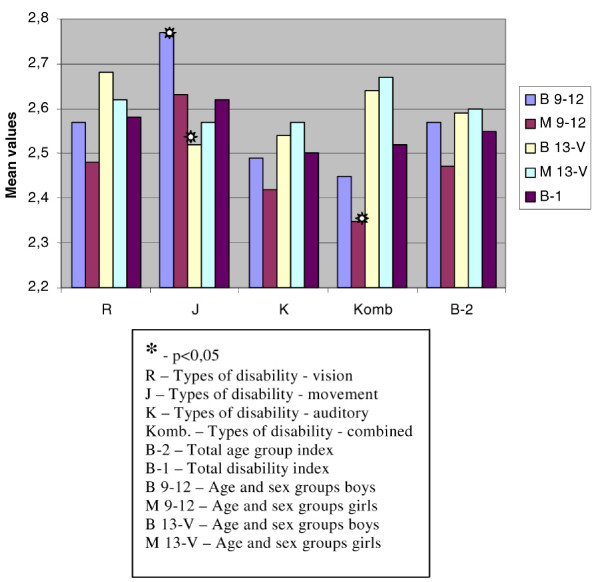
**The self-awareness component of schoolchildren with disabilities by age, sex, and the type of disability**. The relationship between the self-awareness component (SKX) of disabled schoolchildren's feelings towards their disabled peers, and their age, sex (girls, boys), and the type of disability (vision, movement, auditory, combined).

In order to clarify the disabled schoolchildren's evaluation of their possibilities for socialization, we investigated and evaluated the relationship between their self-awareness index (SKX) and their age, sex, and the type of disability [see Figure 7]. The obtained findings showed that total indices (B-1, B-2) did not depend on age, sex, or the type of disability. However, the analysis of these findings in different aspects of age and sex groups and the types of disability showed that SKX in younger boys with movement disability were reliably (p < 0.05) higher, compared to those in their peers with other types of disability, and younger girls with combined and auditory disability especially carefully evaluated their possibilities (positions Komb., and K). The generalization of these findings showed that the total SKX in all groups of respondents was high (2.5 out of 3 points), and tended to increase with age.

**Figure 8:**
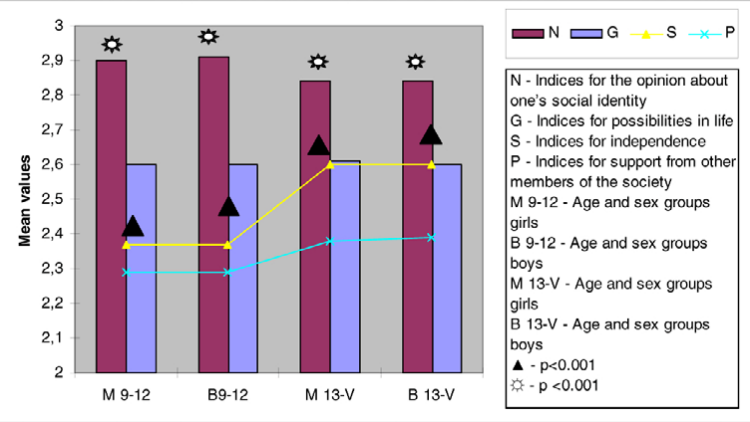
**The self-awareness index of the emotional component of schoolchildren with disabilities by age and sex**. The relationship between the values of the self-awareness index (opinion about one's social identity, possibilities in life, independence, support from other members of the society) of disabled schoolchildren's attitudes, and their age and sex (girls, boys).

The presented data [see Figure 8] show that nearly all the disabled participants of the study, independently of their age and sex, did not tend to segregate themselves from the members of the society without disabilities (position N), and this indicator was higher (p < 0.05) than other indicators of the self-awareness index (positions G, S, and P). In the group of older respondents, the independence indicator was reliably higher (p < 0.001) than that in the younger respondents (position S). Thus, the obtained data showed that the social identity of schoolchildren with disabilities remained high irrespectively of age. Their independence tended to increase with age irrespectively of sex.

## Discussion

Significant attention in our study was paid to the emotions of schoolchildren with and without disability. Models of disabilities analysed in scientific literature differ in their approaches to the nature of the phenomenon of disability and the causes of its development, as well as in the possibilities, ways, and means for the integration of the disabled into the society.

It could have been expected that the attitudes of schoolchildren without disabilities towards their disabled peers in the integrated and the non-integrated comprehensive schools would differ [11]. However, the findings of our study contradicted the contact theory. Scientists who investigated the attitude of schoolchildren with disabilities towards the integration issues stated that the contact theory could only be proven philosophically [12].

Other researchers [13] also noticed that the disabled experience positive emotions when relating to people without disabilities. We think that the fact that schoolchildren with disabilities experienced significantly more positive emotions towards their pears without disabilities than towards other children with disabilities, had a positive influence on their psychological and physical health, and should facilitate their successful integration into comprehensive schools.

We think that it is important that our respondents did not feel fear in relation to each other, and felt safe within their social group [14]. However, sympathy for the disabled was manifested only by schoolchildren with auditory disability.

We determined close relationships between the self-awareness index and the feelings of fear and sympathy, and between the feelings of joy and sympathy and the self-esteem index. This allows for stating that the disabled, being indifferent towards the members of their social group, cannot fully understand themselves and their aspirations. We tend to think that our respondents are oriented towards socialization through suppression.

The suppressive socialization model states that socialization in a disintegrated environment emphasizes obedience, respect for the authority, and external control. During communication, a child is somewhat looked down upon, there is a tendency to command, frequently choosing statements that suppress the child's willingness to answer them. The fact that children with auditory disability exhibited reliably more positive feelings of sympathy and compassion, confirms the predomination of this model in special education establishments.

It is highly important for the learning youth during their maturation period to discover and internalize their identity on the basis of humanistic and global cultural values, the principles of democracy, and universally accepted human rights and freedoms.

The results of our study corroborate the predominant opinion that at present in Lithuania it is still too early to speak about clearly defined state policy with respect to the school-related socialization of the disabled. For this reason we think that disabled children with auditory disability and speaking the state language, whose education has been segregated, should be educated in the integrated way, i.e. together with schoolchildren without physical disabilities, so that the society does not devaluate disabled schoolchildren's capacities.

## Conclusion

The majority of disabled schoolchildren from special schools highly evaluated the indicators of self-esteem and self-concept of the evaluative and behavioural components (from 2.57 to 2.8 out of possible 3.0 pts.) irrespective of their age and sex, which could be considered as a favourable psychological precondition for the development of equal relations in the integrated educational environment.

The level of the self-realization of the disabled increased with their age (p < 0.05), whereas their self-esteem decreased (p < 0.05), and their self-concept remained stable.

Children with vision and movement impairments expressed higher levels of self-realization (p < 0.05) compared to children with hearing and combined disabilities.

## Competing interests

The author(s) declare that they have no competing interests.

## Authors' contributions

All authors read and approved the final manuscript.

LS participated in the formation of the research structure and conducted the main research

AJ participated in the formation of the research structure

GS conducted the pilot study

KJ conducted the pilot study

AL performed the review of literature

## Pre-publication history

The pre-publication history for this paper can be accessed here:
